# Leaving orthopaedic surgical training: the LOST surgeons – a qualitative study exploring why UK trauma and orthopaedic registrars discontinue surgical training

**DOI:** 10.1136/bmjopen-2026-119550

**Published:** 2026-07-13

**Authors:** Joanna Maggs, Hannah James, Julia Gauly, Helen Vint, Homa Arshad, Sarah McMahon, Arabella Scantlebury, Amy Grove

**Affiliations:** 1 Royal Devon and Exeter Hospital, Exeter, UK; 2 University of Warwick, Coventry, UK; 3 Centre for Evidence and Implementation Science, University of Birmingham, Birmingham, UK; 4 Northumbria NHS Trust, Newcastle upon Tyne, UK; 5 Barts Bone and Joint Health, Barts and The London School of Medicine and Dentistry, London, UK; 6 Great Ormond Street Hospital for Children NHS Foundation Trust, London, UK

**Keywords:** ORTHOPAEDIC & TRAUMA SURGERY, SURGERY, QUALITATIVE RESEARCH, MEDICAL EDUCATION & TRAINING

## Abstract

**Abstract:**

**Objectives:**

The study aimed to explore why trauma and orthopaedic registrars decide to discontinue surgical training. Understanding the factors that influence a decision to leave may help to inform changes which enhance the experiences of surgeons and retention of the future workforce.

**Design:**

Qualitative study using semi-structured interviews.

**Setting:**

Between October 2022 and March 2026, interviews were conducted with participants who had left the UK National Health Service (NHS) trauma and orthopaedic specialty training before completion. Reflexive thematic analysis was performed on 1727 min of interview data.

**Results:**

23 respondents were interviewed. Most respondents exited specialty training in the middle years (ST5 and 6). 13 retrained in the NHS and 10 left the NHS entirely. Three overarching themes were identified: (1) ‘The Hidden Curriculum’ describes the presence of unwritten rules, attributes and behaviours which must be met to succeed in training (2) ‘Flawless Care in a Flawed System’ describes surgeons’ responsibility for delivering faultless care while lacking agency to address system failures and (3) ‘Interchangeable and Underappreciated’ depicts the dissonance between trainees’ professional status and their perceptions of being interchangeable.

**Conclusions:**

The loss of surgical registrars from training programmes comes at a high cost to the healthcare system and society as well as to the individual. Understanding what triggers surgeons to leave training allows consideration of how challenges can be modified to prevent further attrition and ensure sustainability of the profession.

**STRENGTHS AND LIMITATIONS OF THIS STUDY**

- The study achieved engagement with a difficult-to-reach professional population.
- The study contains a convenience sample of a mixed population in terms of training grade, gender and ethnicity.
- The data is lacking in the experiences of those doctors in locally employed posts in hospitals and the specialty and specialist workforce.
- Numerous potentially eligible respondents were not willing to participate or be recorded during an interview, which is reflective of the sensitive nature of the topic.

## Background

 In the UK, the shortest route to gaining the certificate of completion of training (CCT) in trauma and orthopaedic (T&O) surgery is 10 years: 2 years of foundation training, 2 years of core training and 6 years of specialty training (ST/registrar). This is generally followed by a year or more of fellowship training. Training in T&O is extremely competitive and achieving a National Health Service (NHS) training post requires investment of significant time and money. The hidden costs of training, beyond tuition fees, are upward of £40 000.^1 2^ Leaving a training post is a life-changing decision for a surgeon.

When a registrar leaves training, the profession loses a member of the surgical workforce; a future consultant surgeon and the capacity to deliver surgical services.^3^ There are around 9000 surgical training posts in the NHS. Between 2016 and 2021, 481 trainees left their NHS posts.^4^ In their study of surgeon attrition rates per year per specialty (limited to 2021), Khalil *et al* reported a 1.52% attrition rate for T&O which was lower than general surgery (3.19%).^4^ The cost to replace NHS doctors who leave is estimated between £1.6 and £2.4 billion.^5^ The loss to patient care is more difficult to measure.^6^ Despite the growing rate of attrition,^4^ little is known about why T&O registrars decide to discontinue their training. This study is the first to address this gap in knowledge and to understand what can be done to ensure the sustainability of the profession.

## Method

We used convenience sampling of ex-registrars who had worked in the NHS between October 2022 and March 2026. Study information was shared via social media channels and via our professional networks. Recruitment was facilitated via UK NHS Training Programme Directors (TPDs), the British Orthopaedic Association and Trainee Association. Eligibility criteria included participants who (1) had a UK training number and started but did not complete orthopaedic training, (2) gave consent to have an interview in English and (3) left training within the past 10 years.

Potential respondents provided written consent. Interviews were conducted via Microsoft Teams (Topic guide online supplemental appendix 1). The topic guide was developed iteratively by the research team, drawing on a review of the existing literature.^6 7^ The development process involved input from all members of the research team, including practising orthopaedic surgeons (JM and HJ) and academics with expertise in qualitative research methodology (AG, JG and AS). Prior to use in the main study, the guide was pilot tested with a small number of individuals who were surgical trainees but were not included in the final study sample. Feedback from the pilot was used to refine the wording, sequencing and scope of the questions.

At the beginning of the interview, respondents were asked to share demographic information. This was followed by an in-depth exploration of their experiences during training and their decision to leave.^8^ Interviews were recorded, transcribed and accuracy checked. No repeat interviews were conducted. Data analysis followed the steps of reflexive thematic analysis; including multiple readings of transcripts and coding data extracts.^9^ Subthemes were developed and summarised as three substantive themes. Deviant cases were identified and interrogated where appropriate. Researcher characteristics include five orthopaedic surgeons (JM, HJ, HV, HA and SMM); three experienced methodologists (AG, JG and AS). Authors who collected data were female (AG and JM) with professional and academic experience of surgical training. Participants were not compensated for their time and took part in the study voluntarily. The study is reported using the Standards for Reporting Qualitative Research.^10^

### Patient and public involvement

Orthopaedic patients and lay members of the public were involved in the initial study design and awarding of the primary research.

## Findings

23 respondents were interviewed. Most respondents exited ST in the middle years (ST5 and 6). Four retrained in different specialties, 11 retrained as general practitioners or allied health professionals (AHPs) and 10 left the NHS (see table 1).

**Table 1:** Respondent characteristics by exit stage and career path

Training stage (n)	Onward career all participants (n)	Gender (n)
Early stage (n=8) ST3/4-PGY5/6	Private sector (n=8) GP training (n=9) Specialty training (n=4) Allied to health non-NHS (n=2)	Female (n=10) Male (n=13)
Middle stage (n=13) ST5/6-PGY7/8		
Later stage (n=2) ST7/8-PGY9/10		

GP, general practitioner; NHS, National Health Service.

Three themes were identified which depict the experience of surgeons in training, the culture of T&O and of working in the NHS: (1) ‘The Hidden Curriculum’ describes the presence of unwritten rules, attributes and behaviours which must be met in order to succeed in training (2) ‘Flawless Care in a Flawed System’ describes the responsibility respondents felt to deliver faultless care while lacking agency to address system flaws and (3) ‘Interchangeable and Underappreciated’ describes the dissonance respondents felt as skilled professionals who were treated as interchangeable workers. Themes are described with illustrative verbatim quotes.

**The hidden curriculum: ‘Yes, Mr.** **Smith. No, Mr.** **Smith. Three bags full, Mr.** **Smith’*****.***

The hidden curriculum is a notion traditionally associated with medical school where students learn a set of opaque lessons.^11^ Our respondents described certain attributes, behaviours and actions that they felt must display to succeed as an orthopaedic trainee. They felt that meeting the expectations of this hidden curriculum was as critical to acceptance and progressing as meeting the expectations of the formal curriculum. However, the hidden curriculum was not openly discussed. Some trainees were felt to have more understanding of it than others, and there was little support in navigating it. Participants described how, when they failed to meet its expectations, they felt demoralised and unsupported:

There are not-fully-verbalised expectations, which takes time to realise. I’m expected to be doing it this way, or I’m expected to be doing that. And you don’t know that until you’ve been [in training] for a while. You feel like you’re making a bad impression, even if you’re trying hard. At each stage of training, as you become more senior, you become more aware of it. It takes time to assimilate.

Certain characteristics were valued over others. These included confidence and dedication at work, being resilient, having a thick skin, being immune to failure and meeting the *“expectation that you are there and seen to be there, even if you’re not necessarily doing anything useful.”* Trainees understood that displaying these traits conferred an advantage:

I’d have to fit into this mould that I kept getting told at ARCPs that I need to fit into. They said you must have points on your elbows, you must be more resilient, less sensitive. I don’t know how to do that. My level of sensitivity, allows me to tune in to how people are feeling, and I wish that that was more valued within orthopaedics

Valued traits were useful to navigate the negative behaviours that were described, by some, as persisting in orthopaedics. These included ‘bullying’ and ‘teaching by humiliation’. Part of the hidden curriculum was understanding the expectation that these behaviours would be navigated or accommodated, rather than challenged:

My sense was, ‘I can’t make a big deal about anything that I’m upset about.’ I can’t even talk to people because I don’t want it to come across as like airing dirty laundry or trying to find excuses for not being a good trainee.

The expectation of compliance was understood across trainees. One participant explained how their rota was repeatedly shown to exceed contracted hours, but they were unable to convince colleagues to escalate concerns:

When I flagged it up to the [other] trainees, we couldn’t get a united front to push it with the union because some people, quote ‘didn’t want to rock the boat’.

Participants were acutely aware that, while meeting the requirements of the formal curriculum was the basis for gaining CCT, meeting the requirements of the hidden curriculum was critical to securing a job as an orthopaedic consultant. This was summarised by one participant who described the reason they didn’t raise concerns about the poor behaviour they experienced:

I should have told someone about it. But you can’t do that whilst you’re a trainee, because you’re on a 12-year job interview at the same time. And, you know, if you do that, your employment prospects have probably gone.

Some participants felt that conforming to stereotyped traits continued to hold currency in orthopaedics. One recipient described themselves as *“a little bit weedier and shorter”* than the stereotypical surgeon. They had been told that they were *“‘too nice to be an orthopaedic surgeon’.”* They suggested that *“to be an orthopaedic surgeon you had to be rude, arrogant, and disinterested in patient care.”* Senior surgeons who may not conform to stereotypes themselves were felt to have nonetheless absorbed them and come to value them:

I still think you have to be hard. You have to be like a man. And that doesn’t help [that] we’ve got female consultants who have that opinion as well*.*

Part of the hidden curriculum required honouring and perpetuating aspects of traditional orthopaedic hierarchies and culture and shouldering any amount of work without complaint. For some, this felt jarring:

There’s an infantilisation and hierarchy in orthopaedics; a lot of old-fashioned behaviour. I’m in my 30s, married, soon to have a child and have to still say ‘Yes, Mr. Smith. No, Mr. Smith. Three bags full, Mr. Smith’ to someone who’s 10 years older than me.

**Flawless care in a flawed system: ‘** **You’re responsible, but you have no control.’**

The data depicted how training programmes were being delivered in failing healthcare systems, yet respondents believed themselves accountable for maintaining infallibility. Poor provision of training was repeatedly acknowledged and tolerated, specifically in relation to adequate operative experience to acquire the necessary competencies to proceed. This was perceived as a failing for which the trainee should personally be accountable, and for which they would be expected to provide a solution:

Even early on, I was thinking, I’m not sure I’m going to get my [operative case] numbers, so I’m going to have to come up with a plan later down the line in training to try and get the numbers up

Participants expressed frustration and resentment at being put in this predicament:

I shouldn’t really have to come in on zero-days to get my numbers, I should really be trained in my actual work time

Repercussions included friction between trainees and negative behaviours:

Everyone was stressed about numbers because there was not enough operating. The senior guys, [who] were about to come off the top, people that had been lovely for years, were elbowing each other out the way for cases, and there was lots of infighting

Trainees felt that trainers perpetuated the sense of personal accountability:

My trainer made me feel like it was my fault, but I don’t see how I could have done things any differently.

Perceived accountability for failure was formalised in the Annual Review of Competency Progression (ARCP). Trainees described raising problems during the ARCP which, in the eyes of the panel, would remain their responsibility to resolve:

My ARCP [panel] made the comment ‘you’ve not done any real cutting yet’. [I said] ‘I’m not sure what you’re expecting. I’ve turned up to all my lists that I’ve been allocated to’.

A lack of resource was seen as something that had to be accommodated by surgeons given increasing constraints on the health system, but not without frustration.

A lot of my time there was no scrubs, there’s no 3/0 sutures, there’s no implants and there’s no theatre staff. Then a dressing or something wasn’t available. I said [to my consultant] ‘Why don’t you say something’? And he actually said, ‘just adapt and get on with it’. And I thought ‘that is almost the problem*’.*

This impacted on service delivery. Participants described how “*theatre efficiency in lots of places is just trending forever downwards”*. One participant explained that theatres routinely over-run which meant that they were always coming into work *“expecting to stay until 10 o’clock, when I knew we could have been finished by 4pm”*

For some, the inability to deliver an adequate service, and their inability to influence this, impacted their job satisfaction:

It just seemed to get worse and worse really. Waiting lists piling up, patients unhappy with the system. We’d used COVID as an excuse for quite a long time, but I felt that, by then, patients, and also us, were a bit bored of making excuses of things, and I felt I wasn’t doing my patients a good enough service.

It was a common narrative that any efforts to improve hospital systems and services would be unsuccessful and unrewarded:

There’s so much frustration trying to make changes: the computer says no, there’s no room for this, there’s no budget for this, there’s no whatever for this. It is so frustrating. I felt like everything was an uphill battle.”

There was a strong sense that the ethical duty doctors feel towards patients was used by the NHS to manage short term service gaps, where more lasting solutions were required. This was summarised by one participant:

We’ve seen a massive hole and we’re going to plaster it with our own goodwill. When you’re a hospital consultant, you can’t really effect change. You know a lot of the things that need to happen to make your department run better, and the care in the department better, but because it’s such a big bureaucratic machine in hospital, it’s so much effort to do that change.

These challenges were problematic for participants, whose role as a surgeon involves making high-risk decisions and taking responsibility

The problem comes as a consultant, when there’s a discrepancy between your responsibility and your control. None of us mind having responsibility. Give us responsibility all day. We’ll take responsibility for people’s lives, for the service, all of it. The problem is, if they say you’re responsible, but you have no control.

**Interchangeable and underappreciated:** ***‘*****A number on a spreadsheet*****’***

Findings highlight how respondents believed that they were nothing more than a ‘number on a spreadsheet’. They described having little agency to affect change because they were perceived as generic trainees and therefore, interchangeable. Respondents’ frustrations about being held accountable for multiple system failings were compounded by having little influence over their own working lives. Several examples were given in which respondents were expected to accommodate challenging commutes, living away from family, moving to multiple hospitals, or missing out on training opportunities, without complaint or at short notice:

I was lined up to do the hip job second time round, but then I got a phone call from the TPD [saying] I need you to go to [a hospital 100 miles away] next month because of someone else’s need. It was looking like I was going to do 6 sites in 6 years. It’s jarring as a trainee being moved around like a piece on the chess board*.*

Requests for alterations or accommodations were often denied and the reasons given were difficult for participants to rationalise:

When you asked for a [location] swap, they would say ‘it’s for your training purposes’. Is it? Why can’t I do hand surgery at this other place? What’s the difference? Why do I have to go two hours away to do this rotation when I could do one that was 45 minutes away?

Respondents recognised that T&O was felt to be a demanding specialty with long hours. However, rotas and hours at work were unnecessarily challenging:

I hope they don’t have this rota anymore. I was on basically a 24 hour every week, and then a 48-hour weekend every fourth weekend and you would then work the next day at the hospital.

I had literally had no break. I didn’t eat at all. My urine output was negligible for 12 hours.

For some respondents the effort they were putting in felt unacknowledged:

For me, it wasn’t working long hours, it was working those long hours and nobody recognising that you’re doing that.

The respondents appeared to suggest that any service failures or under-resourcing would be accommodated through extra efforts of surgeons. One participant explained that when they needed to come off the on-call rota:

The department didn’t hire extra people to cover my nights, it was [up to] the junior tier to cover my nights. They’re getting locum money, but they’re exhausted.

Work-related stress had consequences, including mental health difficulties, burnout and pregnancy complications:

I had a number of miscarriages. Probably related to the extreme stress I was under.

One participant explained that they had a 48-hour weekend shift followed by a full day operating on the Monday. They explained that this was not appropriate or safe. However, on return to work they were met with challenge by their manager:

My supervisor at the time said, ‘I’ve had a complaint about you from the hospital manager. She said you were too lazy to get out of bed’. And, and it actually was a formal complaint against me. It was absolutely unbelievable. I just thought ‘well, there was literally no one who has your back’.

Several participants described feeling institutionalised in the NHS, one respondent reflected on this:

You just get institutionalised, and stop valuing your time and money as a human and employee. I calculated [it cost me] £4800 pounds to work one year, and the difficulty in trying to claim that any molecule of that, the level of barriers was crazy. Compare that [to my employment] now. If I need to come into work the company sort a room and cover costs and sort lunch. They will look after you as a human being. So much of that is just so absent [in the NHS]. In the NHS the level of neglect of its employees in incredible. When you start to work anywhere else you think ‘what on Earth have we been living through this whole time?’.

Working in the NHS more widely was also felt to be undervalued:

I think we all want to feel some level of gratification, we don’t need to have our name in bright lights, we just want to feel that we are valued in a sense that yes, your role here is important.

## Discussion

The study explored why NHS T&O registrars decide to discontinue surgical training. Findings from the analysis of 23 interviews illustrate the factors which influenced a decision to leave, which are distinct from the technical skills of surgical training. The factors we identified are not limited to the NHS context.^12^,^14^ Research in Australia^15^ and the USA^16^ reports similar problems of surgeon attrition including unfavourable work environments, challenges in parenthood, male-dominated cultures and societal pressures.^16^ Liang *et al* work on female surgeons hypothesised that many of the issues which caused women to leave surgery may affect male trainees too.^15^ They described the contributory factors and the small actions that might finally topple the tower, and the contexts in which the blocks are stacked. In our cohort too, participants often described their reasons for leaving as ‘multifactorial’ with the final decision being made when a certain encounter or episode became ‘the straw that broke the camel’s back’.

Our findings from a mixed-gender population provide supportive evidence of this. Respondents stressed that they remained passionate about performing surgery and working with patients. Unfortunately, the systemic organisational problems, and the unattainable behaviours and expected attributes led to their exit. These behaviours and attributes fall outside of the formal curriculum defined by the Intercollegiate Surgical Curriculum Programme in the UK.^17^ In part these attributes were necessary to navigate some of the stereotypical behaviours tolerated within T&O. Excelling at the hidden curriculum requires prolonged exposure and assimilation of a culture that is shared via the cafeteria, changing rooms and the surgeons’ lounge. Registrars who were not comfortable or invited into these spaces found it harder to achieve the expectations of the hidden curriculum and were not considered a successful or committed surgeon. Evidence of a hidden curriculum has been reported during undergraduate medical training, and hypothesised to exist within surgical specialties. In the case of T&O, registrars, expected attributes included displaying confidence and dedication at work, being resilient, having a thick skin, being immune to failure and showing deference to the hierarchy.

As noted in the 2025 Royal College of Surgeons workforce census ‘burnout is widespread’ and attrition risk is rising.^18^ These risks extend beyond T&O and beyond trainees, with census data suggesting 59% of surgical consultants aged 55–59 years plan to retire within 4 years.^17^ The report described inadequate access to theatres which adversely impacted training for 61% of surgical trainees. Participants in our study described their experiences of being held accountable for, and holding themselves accountable for, a high standard of patient care and their frustration at lacking the agency to address the multiple system failures, which made this standard impossible to deliver. Outside of surgery, the workforce retention crisis for medical and AHP is widespread and well documented. We, therefore, anticipate large transferability of our findings and applicability of our data across the NHS Workforce. Our findings described respondents’ experiences of being held to a high standard personally, while the system failures made these standards impossible to achieve and maintain. Respondents appeared comfortable with repeated examination and assessment, but the constraints stemmed from training systems that did not enable them to fulfil their mandatory requirements. We identified that while the T&O registrars were typically motivated senior professionals, they had little agency over the training environment. This appeared to slowly fragment their motivation to overcome challenges, navigate change and respond to setbacks with determination and resilience. Over time, the quest for surgical mastery was paralysed by the lack of autonomy.^19^

The findings illuminate many of the conclusions drawn in the Workforce Census survey by the Royal College of Surgeons of England^20^ which described multiple issues threatening the sustainability of the surgical workforce. The census found that 28% of higher surgical trainees had considered leaving the profession, with lack of training and work-life balance cited as reasons.^20^ A 2026 longitudinal study of Electronic Staff Records for surgeons in England provided by NHS England demonstrated clear and systematic variation in exit and promotion by gender and ethnic group.^21^ Relative to the field average (all surgical specialties), White men remain in surgery at higher rates, with significantly lower exit risk (−35.52%, p<0.001), by contrast, Chinese women exhibit the highest exit risk (97.32%, p<0.001).^21^ Our study was the first conducted to explore the reasons why surgeons exit, and the experience of T&O registrars who had left training. We provide evidence that early warning signs such as lack of training opportunities can lead trainees to quit. These factors are confounded by difficult working cultures and systems which contribute to experiences of burnout and stress. We have found that these challenges begin in the early stages of training and continue long into senior years.

Our study provides insightful evidence which explores how postgraduate medical training is delivered, experienced, and how it might evolve to meet the changing needs of trainees, services and patients.^22^ We have identified clear concerns, frustrations and distress that many respondents faced when undergoing surgical training which led them to relinquish their training number. We also found large variation in respondents’ experiences of training. Some hospitals were named by multiple respondents as institutions where the culture was supportive and learning thrived. Others as places where bullying and discriminatory behaviour are commonplace. There was an unspoken, shared knowledge of what was accepted. The NHS was felt to be inappropriately reliant on the goodwill of doctors to prop up failing systems. Sometimes the extra effort doctors put in was felt to obscure the severity of NHS challenges, allowing them to persist unaddressed. Establishing a culture that recognises and acknowledges the needs of its staff may lead to improved levels of job satisfaction and workforce retention.^23^ Patient satisfaction has been shown to be markedly higher in organisations where staff health and well-being are better.^24^

To help to meet future service requirements and reflect the increased needs for flexible working of trainees we provide recommendations grounded in our data. These recommendations may help to address the factors which contribute to individuals leaving the surgical workforce but also support those surgeons who stay.

1. Prioritise training opportunities in all stages of training programmes and job-planning/mandating time for educational and clinical supervisors to provide training.
2. Ensure minimum standards to support workplace well-being, ranging from dedicated study spaces and flexible schedules to zero-tolerance policies for incivility, bullying and harassment.
3. Ensure independent exit interviews with leavers to understand their reasons for leaving, identify missed opportunities and provide feedback loop to responsible bodies. Exit interviews are commonplace in the corporate world, with three-quarters of companies using them to collect feedback from departing employees.^25^ However, they are rarely held in the NHS. In one study of doctors moving to train abroad, over 96% said they had not been offered an exit interview.^26^ The British Medical Association has called on all NHS employers to conduct and publish the anonymised results of exit interviews through an open access national database to allow job applicants to compare employment conditions.^27^

### Strengths, limitations and areas for future research

A clear strength of our study is the rigorous approach to data collection and analysis and the engagement we were able to achieve with a difficult to reach population. We used convenience sampling to ensure we included a mixed population in terms of grade, gender and ethnicity. However, we included all potential participants who were willing to be interviewed for the study. We have not presented demographic details due to the risk of respondent identification. The demographic characteristics of participants in this study were intentionally limited in scope to protect anonymity, given the relatively small and identifiable population of T&O trainees who have left training. While this was a necessary and ethically justified decision, it means that the potential influence of demographic factors on the experience of training and the decision to withdraw could not be fully explored in this analysis. There is growing evidence that the experience of surgical training is not uniform across all trainee groups. Ethnicity, socioeconomic background, gender and disability status have all been identified as factors that may shape trainee experience, progression and attrition. In T&O specifically, which has one of the lowest proportions of female trainees and consultants of any surgical specialty, gender and intersecting characteristics such as ethnicity and caring responsibilities may compound the structural and cultural barriers that contribute to withdrawal from training. Supportive evidence from the DE FACTO study^27^ demonstrated that differential attainment in T&O training applications and outcomes is associated with demographic and socioeconomic factors, suggesting that attrition is unlikely to be demographically neutral.

Future research should seek to capture a richer demographic profile of trainees who leave T&O, ideally through larger-scale mixed-methods studies or anonymised national datasets, which would allow exploration of whether the themes identified in this study are experienced differently across demographic subgroups and whether they persist across surgical or other medical specialties. Understanding the intersection of demographic characteristics with structural, cultural and personal drivers of attrition is essential to developing targeted, equitable retention strategies that address the needs of all trainees rather than the assumed majority. Missing from our data are also the experiences of those doctors in locally employed posts in hospitals and the specialty and specialist workforce. While these doctors are not in training roles, they may leave surgery for similar reasons to registrars. We encountered numerous potentially eligible respondents who were not willing to participate or be recorded during an interview, which is reflective of the sensitive nature of the topic. Some stated that they could not revisit their traumatic experiences or did not want to be interviewed by anyone representative of the profession. Additional research is needed to examine the extent and prevalence of the challenges we identified and the geographical variation across training regions and countries. This work will need to account for how solutions can be implemented without detriment to the wider health system and patient care.

## Conclusions

The increasing rates of attrition in surgical training in England highlight a problem; however, qualitative research is needed to ascertain the causal factors behind the attrition of surgical registrars to improve training and retention of this highly skilled workforce. This is the first study conducted in UK T&O surgery which explored why registrars decide to discontinue their surgical training. Reasons for leaving training included perceived inadequate training opportunities and inappropriate workplace practices and cultures. Given the moderate to high risk of burnout among medical registrars, the retention of doctors from healthcare systems needs to be an urgent priority for UK governments, health services and employers. Addressing factors which contribute to individuals leaving the surgical workforce will also benefit those surgeons who stay.

## Supplementary material

10.1136/bmjopen-2026-119550 online supplemental appendix 1

## Data Availability

No data are available.
