# Supplementary material for: Leaving orthopaedic surgical training: the LOST surgeons – a qualitative study exploring why UK trauma and orthopaedic registrars discontinue surgical training
Source: BMJ Open. 2026 Jul 13;16(7):e119550. doi: 10.1136/bmjopen-2026-119550 (PMC13365747; doi:10.1136/bmjopen-2026-119550)
Supplement: online supplemental appendix 1 [file bmjopen-16-7-s001.docx]

**Appendix:**

Semi-structured interview topic guide.

1. Can you tell me about your motivation to apply for orthopaedics?

Prompts:

- Can you tell me about the time when you first decided to pursue a career in Medicine?
- What do you think attracted you to orthopaedics?
- Can you tell me about your experience of applying for orthopaedics?

1. Can you tell me about your experiences as orthopaedic trainee?

Prompts:

- What did you like about it?
- What did you not like about it?
- What challenges did you experience and how did you overcome them?
- How did you experience the Workplace Based Assessments?

1. Can you tell me about decision of discontinuing your orthopaedic training?

- Prompts:
- When did you first think of leaving orthopaedic training?
- What were your reasons for deciding to discontinue orthopaedic training?
- Do you feel that the number of Workplace Based Assessments (WBAs) affect your decision?
- How did it feel when you formally quit the training programme?

1. Can you tell me about the time after you left orthopaedic training?

Prompts:

- Have you reflected on your time in orthopaedics since leaving?
- How do you feel about your decision to leave orthopaedic training today?
- Which career path have you decided to take?

1. Can you think of anything that could have been done in the training that would have changed your mind about discontinuing your orthopaedic training?
2. Prompts around support/mentoring/changing regions.
