# Supplementary material for: Leaving orthopaedic surgical training: the LOST surgeons – a qualitative study exploring why UK trauma and orthopaedic registrars discontinue surgical training
Source: BMJ Open. 2026 Jul 13;16(7):e119550. doi: 10.1136/bmjopen-2026-119550 (PMC13365747; doi:10.1136/bmjopen-2026-119550)
Supplement: Supplementary file 2 [file bmjopen-2026-119550.reviewer_comments.pdf]

PEER REVIEW HISTORY

BMJ Open publishes all reviews undertaken for accepted manuscripts. Reviewers are asked to complete a checklist review form and are provided with free text boxes to elaborate on their assessment. These free text comments are reproduced below.

ARTICLE DETAILS

Title (Provisional)

Leaving Orthopaedic Surgical Training: the LOST surgeons: a qualitative study exploring why UK Trauma and Orthopaedic registrars discontinue surgical training.

Authors

Maggs, Joanna; James, Hannah K; Gauly, Julia; Vint, Helen; Arshad, Homa; McMahon, Sarah; Scantlebury, Arabella; Grove, Prof Amy

---

VERSION 1 - REVIEW

Reviewer 1

Name Coates, Laura

Affiliation Oxford Brookes University Faculty of Health and Life Sciences, NDORMS

Date 31-Mar-2026

COI None

This is a very timely and helpful paper addressing T&O trainees leaving training in the UK. The interviews highlight key themes explaining reasons for people leaving training, which, as the authors highlight, has important ramifications for NHS training and service provision. I have a few comments

1. It is mentioned that PPI was involved - were these patients or other T&O trainees given that the focus of the research is on workforce?

2. The study mentions that 481 surgical trainees left training over the last 10 years. How many were T&O? I wonder what proportion were represented in the sample size of 23?

3. 10/23 trainees interviewed were women. Although I do not have the numbers of T&O trainees in the UK, I imagine this is disproportionately more women compared to the overall training numbers. Although this is touched upon a little in theme 1 (hidden curriculum), it would be nice to understand this issue a little more. Or is this just based on the purposive sampling? The discussion mentions that some potential participants declined - how many declined?

4. The second theme (flawed care) seems particularly to be related to training and craft training in particular - should this come out in the name of the theme?

**Reviewer**	**2**
**Name**	**Leighton, Paul**
**Affiliation**	**University of Nottingham, Division of Primary Care**
**Date**	**15-Apr-2026**
**COI**	**None**

I enjoyed reading this paper and found its results interesting.

1. It would be helpful to see your 'purposive sampling' frame and also to reflect on the extent to which you are successful in achieving this - i.e. did you interview the types and variety (?) of participants that you intended, can you show how your sample is purposive and not simply pragmatic/convenient. Table 1 shows who you interviewed, a table showing who you thought you should interview would be interesting.

You note the challenge that too much demographic information might effectively identify the participants, but i wonder if you might reflect in your discussion/limitations upon the role that other demographic characteristics might play in the experience of training / withdrawal from training (ethnicity/ background etc.).

2. Can you identify the speakers of the interview extracts presented. Initials are used in a small number of occassions but most quotes are unidentified. At least it would be helpful to feel confident that quotes are from multiple partipants. Would it be possible to use identifiers that indicated something about their experience (i.e. use table 1 to categorise participants) - might this demonstrate some further patterns in the data? You present your findings/discussion so as to show three broad, overarching narratives of experience, it would be interesting to unpick variations therein and to understand these.

3. Could you comment on how typical TO might be in relation to other specialties? are you findings relevant beyond TO. or is TO distinctive in some way to other specialities? what evidence about the reason for training withdrawal exists in other specialties, are there lessons to learn from these?

4. you focus narrowly on 3 topics, each of which demonstrate the culture of TO training and those settings where it takes place. The topic guide suggests that other aspects might have been considered - reflections on the decision for example.

Could you offer a short commentary at the beginning of the results which reflects upon the breadth and range of topics discussed prior to your more focused presentation.

VERSION 1 - AUTHOR RESPONSE

Reviewer 1
This is a very timely and helpful paper addressing T&O trainees leaving training in the UK. The interviews highlight key themes explaining reasons for people leaving training, which, as the authors highlight, has important ramifications for NHS training and service provision.
Thank you for recognising the importance of this research.
1. It is mentioned that PPI was involved - were these patients or other T&O trainees given that the focus of the research is on workforce?
No, the PPI group were orthopaedic patients and lay members of the public. We have added the words "orthopaedic" and "lay" to page 4 line 254.
2. The study mentions that 481 surgical trainees left training over the last 10 years. How many were T&O? I wonder what proportion were represented in the sample size of 23?
Unfortunately, despite our best efforts, the research team could not access data on the breakdown of surgical trainee attrition by specialty (including Trauma & Orthopaedics) from national NHS or GMC workforce figures. We contacted the Joint Committee on Surgical Training to locate specialty-specific reports, but these were not available. Any individual reports from trainees/trainee programme directors could not be shared due to GDPR restrictions. The figure reported in our manuscript is the most recent in the public domain (Khalil and colleagues4. 10.1016/j.surge.2022.09.001) In this work, the authors submitted freedom of information requests to Health Education England. If we assume T&O's share of the surgical trainee workforce is ~26% (our unpublished evidence personal communication) as a proxy, we can assume 125-130 T&O trainees left training over the 2016-2021 period. However, we chose not to include this approximation in the absence of specialty-specific published totals.
We have included the 5-year T&O average from Khalil and colleagues noting the limitation that data runs to 2021. For absolute clarity we have added the citation reference to the end of the section and to the end of the sentence. Page 3 line 201.
We have also cited a recent longitudinal analysis of staff outcome data (published April 2026 whilst our paper was under review). This gives summery statistics about attrition and gender across all surgical specialities.
"A 2026 longitudinal study of Electronic Staff Records for surgeons in England provided by NHS England, demonstrated clear and systematic variation in exit and promotion by gender and ethnic group.22 Relative to the field average (all surgical specialities), White men remain in surgery at higher rates, with significantly lower exit risk (−35.52 per cent, $p < 0.001$), by contrast, Chinese women exhibit the highest exit risk (97.32 per cent, $p < 0.001$)."
Additional citation has been added to the reference list.
22. Woodhams C, and Parnerkar I. (2026), Institutional Logics and Relational Inequality in UK Surgery: Demographic Dominance and the Uneven Governance of Careers. J. Manage. Stud.. https://doi.org/10.1111/joms.70107

3. 10/23 trainees interviewed were women. Although I do not have the numbers of T&O trainees in the UK, I imagine this is disproportionately more women compared to the overall training numbers. Although this is touched upon a little in theme 1 (hidden curriculum), it would be nice to understand this issue a little more. Or is this just based on the purposive sampling?
We agree with the reviewer. We also believe women are disproportionately leaving the T&O speciality when compared to men and are notoriously underrepresented in research. However, we do not have access to quantitative evidence to support our supposition and therefore, did not include it specifically in the research findings.

As outlined in response 2 above, data which details the demographic information of specialty-specific leavers is not available in the public domain. The paper by Khalil and colleagues4 did not include a breakdown of the demographic details of leavers only total numbers.
Our sampling aimed to ensure a balance across genders in the interviews. In response to reviewer 2, we have changed the wording to convenient sampling rather than purposive sampling.

4. The discussion mentions that some potential participants declined - how many declined?
Seven declined to participate in the interviews. 1 declined to be recorded.

5. The second theme (flawed care) seems particularly to be related to training and craft training in particular - should this come out in the name of the theme?
Thank you for this observation. We have edited the title of this theme as follows
Flawless Care in a Flawed System - "You're responsible, but you have no control."
Page 6 line 373.

Reviewer 2

I enjoyed reading this paper and found its results interesting.

Thank you.

1. It would be helpful to see your 'purposive sampling' frame and also to reflect on the extent to which you are successful in achieving this - i.e. did you interview the types and variety (?) of participants that you intended, can you show how your sample is purposive and not simply pragmatic/convenient. Table 1 shows who you interviewed, a table showing who you thought you should interview would be interesting.
On reflection, we agree with the reviewer that we did perform convivence sampling. Whilst we actively sought to balance genders/ethnicities in out sample, we did not refuse to interview any potential participants who responded to our recruitment campaign.
We have corrected this in the manuscript throughout.

2. You note the challenge that too much demographic information might effectively identify the participants, but i wonder if you might reflect in your discussion/limitations upon the role that other demographic characteristics might play in the experience of training / withdrawal from training (ethnicity/ background etc.).
Thank you, we agree and have expanded our discussion on page 11 line 646-669.
"The demographic characteristics of participants in this study were intentionally limited in scope to protect anonymity, given the relatively small and identifiable population of T&O trainees who have left training. While this was a necessary and ethically justified decision, it means that the potential influence of demographic factors on the experience of training and the decision to withdraw could not be fully explored in this analysis. There is growing evidence that the experience of surgical training is not uniform across all trainee groups. Ethnicity, socioeconomic background, gender, and disability status have all been identified as factors that may shape trainee experience, progression, and attrition. In T&O specifically, which has one of the lowest proportions of female trainees and consultants of any surgical specialty, gender and intersecting characteristics such as ethnicity and caring responsibilities may compound the structural and cultural barriers that contribute to withdrawal from training. Evidence from the DE FACTO study25 demonstrated that differential attainment in

T&O training applications and outcomes is associated with demographic and socioeconomic factors, suggesting that attrition is unlikely to be demographically neutral.
Future research should seek to capture a richer demographic profile of trainees who leave T&O, ideally through larger-scale mixed-methods studies or anonymised national datasets, which would allow exploration of whether the themes identified in this study are experienced differently across demographic subgroups and whether they persist across surgical or other medical specialties. Understanding the intersection of demographic characteristics with structural, cultural, and personal drivers of attrition is essential to developing targeted, equitable retention strategies that address the needs of all trainees, rather than the assumed majority."

Grover S, Raj S, Spazzapan M, Russell B, Bola H, Biju N, Malde S, Fleming S, Vig S. DiffErential attainment and Factors AssoCiated with Training applications and Outcomes (DE FACTO) for general surgery applications in the UK: retrospective study. BJS Open. 2024 Dec 30;9(1):zrae166. doi: 10.1093/bjsopen/zrae166. PMID: 39883739; PMCID: PMC11780877.

3. Can you identify the speakers of the interview extracts presented. Initials are used in a small number of occassions but most quotes are unidentified. At least it would be helpful to feel confident that quotes are from multiple partipants. Would it be possible to use identifiers that indicated something about their experience (i.e. use table 1 to categorise participants) - might this demonstrate some further patterns in the data?

You present your findings/discussion so as to show three broad, overarching narratives of experience, it would be interesting to unpick variations therein and to understand these.]
Thank you for this suggestion and we appreciate the reviewer's recognition to preserve anonymity and confidentiality of our participants; particularly as noted above that our participants represent a small and potentially identifiable sub sample of a surgical speciality. We do, however, recognise the need to provide assurance for quality purposes to demonstrate that our quotations have been selected from across our sample and that we have not relied on a single participant transcript. Given the small number of participants in the paper we do not feel confident, on ethical grounds, that participants would not be identified.
We agree with the reviewer that it would be interesting to understand whether there are any variations or divergences in opinion/experiences according to participant characteristics (hospital type, training level, gender, ethnicity). However, whilst we proactively sought to explore this in our analysis, we did not identify any noteworthy variations. This would be an interesting thread to explore in further larger qualitative studies, where you could explore this across multiple specialties. We expect that our inability to identify this variation is a product of this work being undertaken with trainees within a single specialty and so the numbers were too small to identify any meaningful patterns that we could not attribute merely to chance. It is also important to note that these variations were not raised by participants themselves, which one may have expected to see.

4. Could you comment on how typical TO might be in relation to other specialties? are you findings relevant beyond TO. or is TO distinctive in some way to other specialities? what evidence about the reason for training withdrawal exists in other specialties, are there lessons to learn from these?
Thank you, we have provided the additional comment below to demonstrate that this is not a "problem" occurring only in T&O and reference the recent workforce report by the RCS. See page 9 lines 567-570.

“As noted in the 2025 Royal College of Surgeons workforce census “burnout is widespread” and attrition risk is rising.18 These risks extend beyond T&O and beyond trainees, with census data suggesting 59% of surgical consultants aged 55-59 years plan to retire within four years. 18 Outside of surgery, the workforce retention crisis for medical and allied health professionals is widespread and well documented. We therefore anticipate large transferability of our findings and applicability of our data across the NHS Workforce”.

RCSE. (2025). Advancing the Surgical Workforce: 2025 UK Surgical Workforce Census Report. Available at https://www.rcseng.ac.uk/news-and-events/media-centre/press-releases/workforce-census-2025/ Accessed 19/05/2026

4. you focus narrowly on 3 topics, each of which demonstrate the culture of TO training and those settings where it takes place. The topic guide suggests that other aspects might have been considered - reflections on the decision for example.
Could you offer a short commentary at the beginning of the results which reflects upon the breadth and range of topics discussed prior to your more focused presentation.

For the sake of brevity and to conform to journal word count restrictions we have chosen to present our data through this structure. However, this decision was predominantly determined by our interpretive analysis. Our thematic analysis is presented so as to answer the research question, and to ensure that we are “telling the story, rather than reporting the facts.” (Sheard et al 2022). Whilst indeed we could add a more descriptive account of all the topics that were discussed during our interviews we feel this would detract, rather than add to our over-all messaging and would result in a lower-quality, more list-based analysis and reporting style.

Sheard, L. (2022). Telling a story or reporting the facts? Interpretation and description in the qualitative analysis of applied health research data: A documentary analysis of peer review reports. SSM-Qualitative Research in Health, 2, 100166.

VERSION 2 - REVIEW

Reviewer 1

Name Coates, Laura

Affiliation Oxford Brookes University Faculty of Health and Life Sciences, NDORMS

Date 22-May-2026

COI

All of my comments addressed fully.

Just in relation to the other reviewer's comment. Could quotes be attributed to patients just by number to show the breadth of different participants quotes? That would avoid any concerns on anonymity (which I think is justified). Just state patient 2 or patinet B?

Reviewer 2

Name Leighton, Paul

Affiliation University of Nottingham, Division of Primary Care

Date 04-Jun-2026

COI

Thank-you for considering and reponding to my comments.

Your additional thoughts about demographic factors are welcomed and your identification of this as a potential area of future work is well-made.
